# Peer-learning reviews to improve Gauteng community-oriented primary care: Findings from AitaHealth™-enabled ward-based outreach teams

**DOI:** 10.4102/phcfm.v12i1.2155

**Published:** 2020-03-05

**Authors:** Tessa S. Marcus, Elizabeth Reji, Sanele Ngcobo

**Affiliations:** 1 Department of Family Medicine, School of Medicine, Faculty of Health Sciences, University of Pretoria, City of Tshwane, South Africa; 2 Department of Family Medicine, School of Medicine, Faculty of Health Sciences, University of the Witwatersrand, Johannesburg, South Africa; 3 Department of Family Medicine, Faculty of Health Sciences, University of the Free State, Bloemfontein, South Africa; 4 Clinical Associate Programme, Department of Family Medicine, School of Medicine, Faculty of Health Sciences, University of Pretoria, City of Tshwane, South Africa

**Keywords:** Peer learning, Quality improvement, ICT-enabled community-oriented primary care, Community healthcare, Primary health re-engineering

## Abstract

**Background:**

In 2016 the Gauteng Department of Health engaged University of Pretoria Family Medicine to provide` education, training and information and communication technology support for the phased scale-up of ward-based outreach teams (WBOTs) through community-oriented primary care (ICT-enabled COPC). As in all service delivery, quality assurance is essential. In contemporary best practice, it brings together peer-to-peer learning and quality improvement (QI) in what is termed here as peer-learning reviews (PLRs).

**Aim:**

To assess implementation fidelity and assure the quality of community-based healthcare services.

**Setting:**

This study was conducted in two districts of Gauteng province, South Africa.

**Methods:**

A 3-day PLR of paired WBOTs was conducted by multi-disciplinary teams of academics, partners and site-selected healthcare practitioners. Guided by a benchmark survey distilled from the seven COPC practice elements, they conducted individual interviews, accompanied WBOT members in field and facilitated solution-focused peer exchange workshops with all participants.

**Results:**

At all sites there was clear evidence of achievements and practical challenges with respect to mapping; support, networks and partnerships; infrastructure and functional equipment; work integrated learning; data and service activities; and performance status and management. Methodologically, PLRs supported inclusive, context-specific learning for all along the healthcare service pathway. They generated action plans derived from shared understanding and joint decision-making.

**Conclusion:**

The PLRs and the implementation results demonstrate the importance of structuring learning into service and research. Both helped develop participants’ abilities to understand what they do, do their work, grow their sense of self-worth and improve their relationship with others.

## Background

The success of healthcare reform in South Africa^1^ hinges on services that can respond to the complexity of health in context.^1,2^ A system that is functional, provides quality care and is universally accessible needs to integrate healthcare from the home to and from health facilities through levels of services and across public, private, non-governmental and traditional sectors.^3^ Guided by community-oriented primary care (COPC), services should be rendered by multi-disciplinary teams of healthcare professionals and workers informed by the best available practices and knowledge. Delivery should be geographically focused, person- and family-centred, comprehensive, generalist and equitable.^4^

In COPC, the community-based service delivery pathway involves identifying the community, defining a team’s collective and individual practice boundaries, identifying the population as well as the resource asset base within it and then providing services. Workplace learning is structured into the service pathway through information technology (IT) as well as organised curriculation in order to continuously develop the individual and collective capacity of healthcare providers.

As in all service delivery, quality assurance is essential. In the health setting, quality improvement (QI) usually focuses on aspects of clinical care and system performance.^5^ Methodologically, QI initiatives are professional and researcher-led projects that are commonly conducted internally within units, components or teams. In COPC, a quality assurance peer-learning review (PLR) process has been developed. Informed by peer-to-peer learning initiatives,^6^ peer and learning are explicitly included in the terminology. Peer-learning reviews are processes and activities that bring learning and QI together around areas of service practice to support implementation fidelity and improve service quality. Peer-learning reviews also respond to the need to make the process internally productive, to encourage QI as ongoing and integral to performance rather than as stand-alone, once-off activities and to remove any audit or inspection connotations.

At the end of 2016, the Gauteng Department of Health engaged the University of Pretoria’s Department of Family Medicine (UP-DFM) to provide education, training and IT support for the phased scale-up of ward-based outreach teams (WBOTs) through information and communication technology-enabled community-oriented primary care (ICT-enabled COPC).^7^ Envisaged as the first of several waves, implementation began in 2017 with a cohort of up to 40 teams (eight teams per district) selected from existing functioning WBOTs where community health workers (CHWs) had completed the national Department of Health’s 10-day Phase 1 CHW training programme.

AitaHealth™ is a custom-built, gadget and web electronic data and human resource management support IT platform. It enables information, service and relationship continuity, as well as service and performance management.^9^

This article presents the results of PLRs of ICT-enabled COPC implementation in two districts of Gauteng province. The PLRs were undertaken between 03–05 October 2017 (Site 1) and 12–14 October 2017 (Site 2) with 16 WBOTs using AitaHealth™. They had been operational for between 6 and 3 months (Site 1: April–September; Site 2: July–September), during which time they had registered 5208 and 2096 households and 17 420 and 6312 individuals, respectively.

## Methods

### Study design

The PLR process is a structured undertaking designed to develop individual, team and management ability to self-assess and improve the quality of their practice. The purpose of PLRs is to monitor the fidelity of implementation of community-based services in a systematic way, identify best practices and critical challenges, and develop adaptive action plans through reflection to improve practice. The methodology has been developed and extensively tested by the ‘Cities exchanging on local energy leadership’ (CASCADE) and several other European Union projects, as well as in South Africa.^6^

### Setting

The study was conducted in West Rand and Ekurhuleni districts of Gauteng province, South Africa. The districts differ significantly in population size and density (Ekurhuleni 1609/km^2^ and West Rand 200/km^2^) although their demographic characteristics are similar. In 2016, Ekurhuleni had a population of some 3.38 million people, with 52% of 1.3 million households having two or fewer inhabitants. West Rand had an estimated population of 838 600 people living in 330 572 households, with 51% having two or fewer inhabitants.^8^ Neither site has the requisite organisational arrangements to support fully functional community-based service delivery.

### Selection of participants

The data reported in this article come from a PLR of 16 teams made up of 16 enrolled or professional nurse team leaders (eight outreach team leaders [OTLs] per site) and 139 CHWs (71 at Site 1 and 68 at Site 2) Table 1 describes the field work process.

**TABLE 1:** Fieldwork process – Peer-learning review.

Day		PLR Team A	Team B site visit
		Activities	
Day 1	Constitute a peer-learning review team – include the Team A team leader, a CHW, a health professional, master trainer or other manager	Go over the purpose of the review.	Complete individual benchmark surveys with the host team leader, facility manager and /or CHWs
		Clarify and address expectations	Accompany team members as they go about their service work tasks, using the benchmarks to guide in-field focus
		Designate in-field activities Post-field – reflect and prepare written notes	Discuss issues and share insights with their host team leaders and other managers
		**PLR Team B**	**Team A site visit**
		**Activities**	
Day 2	Constitute a peer-learning review team – include the Team B team leader, a CHW, a health professional, master trainer or other manager	Go over the purpose of the review	Complete individual benchmark surveys with the host team leader, facility manager and/or CHWs
		Clarify and address expectations	Accompany team members as they go about their service work tasks, using the benchmarks to guide in-field focus
		Designate in-field activities Post-field – reflect and prepare written notes	Discuss issues and share insights with their host team leaders and other managers
		**Peer exchange workshop**	
Day 3	Participants	All members of Team A and Team B Facility and subdistrict/district managers External reviewers
	Activities	Prepare findings (Team A and Team B separately) Present findings (Team A and Team B) to plenary Facilitate plenary discussion Facilitate prioritisation of issues/actions (External reviewers) Facilitate plan of action (External reviewers/WBOT coordinator)
	Focus	Challenges Best practices Possible solutions
	Outcome	Identified priorities (Teams A and B) Agreed plan of action (Teams A and B)

CHW, community health worker; PLR, peer-learning review; WBOTs, ward-based outreach teams.

At Site 1, 12 participants completed the benchmark guide as a survey: five CHWs, three facility managers and four OTLs. There were 31 participants in the peer exchange workshop: nine CHWs, one family physician, 10 OTLs, two WBOT coordinators and nine members of the University of Pretoria/International Training and Education Center for Health (UP-I-TECH) team. At Site 2, 11 participants completed the benchmark guide as a survey: four CHWs, two facility managers, four OTLs and one WBOT coordinator. There were 33 participants in the peer exchange workshop: 13 CHWs, one family physician, nine OTLs, three WBOT coordinators and seven UP-I-Tech team members.

### Data collection

To understand the practices and assess progress in WBOT implementation, key elements of the COPC pathway were translated into seven COPC practice benchmarks. These are (1) mapping; (2) support, networks and partnerships; (3) infrastructure and functional equipment; (4) work integrated learning; (5) data; (6) service tasks and activities; and (7) performance status and management approach. The benchmarks were developed into an interview guide, which was used as a tool to collect the data.

The PLR was conducted by multi-disciplinary teams of academics (UP-DFM), partners (I-Tech-South Africa) and site-selected healthcare practitioners (district/subdistrict/facility managers, clinicians, team leaders and CHWs).

Data were collected by using the benchmark interview guide. It was completed individually as a survey (face-to-face or self administered) by up to three people per team on the day of the site visit, either prior to or after going out into the field. The benchmark guide was also used to direct field visit interactions and observations. Field notes were taken by reviewers, collated, shared and cross-referenced with the team, and developed into presentations for discussion at the peer-learning exchange workshop. At the workshop, feedback was noted and incorporated into the data where it was used to support reflection and planning. A report was prepared for and shared with the WBOT coordinators of the respective sites.

### Data analysis

The results of the PLR were collated and analysed using the benchmark guide as an analytic framework. The framework is organised thematically. Data recorded on paper were captured electronically on Microsoft Word or on Qualtrics® survey and analytic software.

## Ethical considerations

Informed consent was obtained from all participants involved in the benchmark surveys, field work and peer exchange workshops. Confidentiality and privacy were strictly adhered to and no names of individuals were recorded or made known in the collection or reporting of information. Data analysis was performed during and after collection. The study did not involve patients. The study was granted ethical clearance by the Research Ethics Committee of the Faculty of Health Sciences, University of Pretoria (Ethics reference number: 102/2011).

## Results

Drawing on the benchmarks in the guide, the results are presented thematically as follows: mapping; support, networking and partnerships; infrastructure and functional equipment; learning in the workplace; activities, service organisation and supervision; and recommendations and forward planning.

### Mapping

In the implementation of COPC, mapping is used to identify WBOT community practice areas, allocate CHWs to households and identify local organisations and institutions within them.

#### Community healthcare practice areas and households

In terms of mapping community healthcare practice areas, teams all indicated that they used and created maps of their areas and households both as a desktop activity and through physical mapping. They used maps to allocate teams and CHWs to physical areas and households. The mapping of CHW practice area households itself was mostly done using hand-drawn maps. Mapping was used to verify available settlement information, while maps were used to support in-field service management (Table 2).

**TABLE 2:** Maps and mapping.

Household mapping	
**Creation and purpose**	‘The team used maps from the housing department. They also used maps that they created on their own’. (S1–6, CHW, M, 04/10/2017)
	(The CHW) collected the map from the library, got addresses and then made own maps. (S2–2, CHW, F, 10/10/2017)
	It helps to identify the demarcation area of each and every CHW to organise services. (S1–10, OTL, F, 04/10/2017)
	We count houses by using a notebook and by using house numbers. We draw the map physically. (S1–1, CHW, F, 03/10/2017)
	Community health workers made a walk about. [They] counted households allocated to them. After the counting (they) took a large sheet a paper and a pen and drew allocated households and streets and named them. (S2–5, OTL, F, 10/10/2017)
	Maps helped to verify where people stay and therefore to find people. (S2–2, CHW, F, 10/10/2017)
	Maps are used for allocations and tracing purposes, to walk easily and not get lost. If a team member requires assistance from a team leader, it becomes easier to give him or her directions through street names and landmarks. (S1–9, OTL, F, 03/10/2017)
**Operational challenges**	(There are) lots of houses which do not have addresses. As a result there is no sequence, and we only rely on hand-drawn maps as the municipal map misleads. (S2–3, CHW, F, 11/10/2017)
	When there is an open space, the numbers of households get mixed up. (S1–2, SN, F, 03/10/2017)
	Physically we have to count them due to a lot of shacks. (S2–5, CHW, F, 11/10/2017)
	The number of households within the allocated area increases after an area is mapped. (S1–6, CHW, M, 04/10/2017)
	The next time when they come, the shacks have increased. (S1–4, CHW, F, 04/10/2017)
	The previously registered households have relocated, especially people from the squatter camp(s). (S2–2, CHW, F, 10/10/2017)
	Security guards refuse to open for them in flats. What they do is to count the building. (S1–3 CHW, F, 03/10/2017)
**Management challenges**	The map from library was taken back. The hand-drawn maps are kept with the OTL. (S2–2, CHW, F, 10/10/2017)
**Organisational mapping and LISA**	
**Purpose, use and challenges**	(We) identify stakeholders (Nthirisano), SASSA, Ward Councillors and CDWs (community development workers). We refer through a social worker based in the facility, who is available on every Thursday. (S1–1, CHW, F, 03/10/2017)
	(LISA is there)… to be able to know who they will be working with (and) for all stakeholders to utilise their skills in improving WBOT work. (S2–3, CHW, F, 11/10/2017)
	She identifies tuck shops, schools, GPs, old age home and any other structures like churches. She then asks the contacts for all the structures in the area. (S2–1, CHW, F, 10/10/2017)
	LISA makes the work of WBOT easier. …to refer households who need food parcels, children to one NGO to help with homework,…(and) to Sunday schools in churches, to help them keep busy, away from streets. (S2–2, CHW, F, 10/10/2017)
	(I have) identified and established relationships with stakeholders. What is left is the paperwork. (S1–3, CHW, F, 03/10/2017)

CHW, community health worker; OTL, outreach team leader; WBOT, ward-based outreach teams; GP, general practitioner; SASSA, South African Social Security Agency.

Ward-based outreach teams encountered several mapping and household verification challenges. Through the process of mapping the teams found the numbers attributed to stands on municipal maps to be non-sequential and almost always out of date. They found buildings were put to multipurpose use. A team leader at Site 1 said they found tuckshops converted to rooms to accommodate tenants, for example. They discovered that people migrate into areas as well as in and out of households after CHWs have physically verified households and tenants. Community health workers also had problems of access, particularly locked gates, dogs in yards and people away at work during the week, as well as security guards.

Amongst the teams there was some variation in terms of where maps were stored and displayed. Cadastral maps were either kept at the facility or returned when they had been borrowed. Hand-drawn maps were sometimes kept by the CHWs who created them. Otherwise, these too were kept by OTLs at facilities, either in files or, more rarely, on display on the facility office wall.

#### Mapping organisations and institutions

The mapping of organisations in communities is also part of initial operationalisation as well as the ongoing functioning of COPC. Organisational mapping was used to identify stakeholders and partners. As with households and individuals, CHWs found service providers also moved around and were not permanent.

Teams are expected to use the ‘local institutional support assessment’ (LISA) tool to help determine the institutional community asset base. However, most teams said that they either had not used the tool at all or had used it sporadically. This was partly because they did not have copies of the paper-based instrument or partly because they saw the tool as ‘paperwork’, a bureaucratic formality rather than as something useful to them. Those who had used the LISA tool said they found it useful.

### Support, networking and partnerships

Creating and maintaining relationships are important for the establishment and ongoing practice of community-based service delivery. In the PLR, participants described their relationships with individuals, families and communities, as well as with healthcare and other service providers (Table 3).

**TABLE 3:** Support, networking and partnerships.

Individuals, families and communities	
**Relationship quality and management**	We have a nail and a finger relationship. (S1–1, CHW, F, 03/10/17).
	(We) are granted access in households (and have a) good relationship with families. They are willing to be given health education. (S2–5, CHW, F, 11/10/17)
	(CHWs) have a good working relationship. Families rely on them. They send people to call them when there is a problem or help is needed. (S1–4, OTL, F, 04/10/2017)
	Families open the door for us but refuse to give information… at times an individual in the house may differ and will have a negative attitude. (S1–9, CHW, F, 03/10/17)
	Sometimes they are busy and you have to wait for them. Some are not welcoming. (S2–1, CHW, F, 10/10/2017)
	Community members demand food parcels before registration. (S1–4, OTL, F, 04/10/2017)
	After they chase CHWs from their houses, they come to the clinic to report that (we) did not come to DOT them. (S2–3, CHW, F, 11/10/2017)
	(I) had a patient who did not want to be assisted. (I) persuaded him after he had defaulted for more than 2 years. He is currently on track and adhering. (S2–3, CHW, F, 11/10/2017)
	There are those that are resistant. They give a wrong address. Some refuse to be registered. Some chase us away. Over time, however, after they witness WBOT work, they seek help. (S2–2, CHW, F, 10/10/2017)
**Local authorities, ward and clinic committees**	
**Relationship quality**	During events the Councillor is notified and invited in events. In meetings (he) talks with the community to be understanding when CHWs visit households. (S2–5, CHW, F, 11/10/17)
	There is a very good relationship with the ward councillor. The councillor provides WBOT slots to address issues during community meetings. (S1–6, CHW, M, 04/10/2017)
	We meet her and also get information about community meetings from her. (S1–10, OTL, F, 04/10/2017)
	One member of WBOT serves on the (clinic) committee. Challenges are presented to her and she also gives feedback. (S1–3, CHW, F, 03/10/2017)
	We met (the clinic committee) once to sort out a challenge about mapping for WBOT and the community in that ward did not know WBOT and we were able to sort the issue. (S2–10, OTL, F, 10/10/2017)
**Heath services**	
**Relationship quality and challenges**	As the community will not allow CHWs in if they are not identified, we have created our own name tags for WBOT so that the community can see them. (S1–5, FM, F, 04/10/2017)
	I think they sometimes feel they are not part of the staff as the order of reporting is not my sole responsibility, as that role is mostly done by OTLs. (S1–5, FM, F,04/10/2017)
	They are not involved in time, (only) when the team is overwhelmed. (S2–4, OTL, F, 10/1117)
	CHWs are made to divide their time between facility and community work. (S2–3, CHW, F, 11/10/17)
	When there is a shortage, the WBOTs don’t get released. (S1–2, OTL, F, 03/10/2017)
	After two (o-clock) team leaders work in the clinic. They cannot do their administration work if they are sitting in front of a gadget. (S1–9, OTL, F, 03/10/2017)
	We are made to queue for files and treatment whereas they agreed on prepacked packages. We wait before being attended. (S1–3, CHW, F, 03/10/2017)
	(There is) no office space where we [could] meet. At times we meet behind structures with no chairs or we meet at the waiting areas in a clinic. (S1–9, OTL, F, 03/10/2017)
	CHWs should be treated like clinic staff. (S2–11, FM, F, 10/10/2017).
	They (HAST) provide us with the list of defaulters to trace. (S2–10, OTL, F, 10/10/17)
**Social development and third-sector partners**	The clients are referred to social development. We meet with the social workers at least once a week. (S1–2, OTL, F, 03/10/2017)
	The NG Kerk provides patients with food parcels while waiting for social development department. (S1–5, FM, F, 04/10/2017)
	We refer elderly patients for bed bath. They also help them to do exercises. (S1–10, OTL, F, 04/10/2017)
	They provide wheelchairs and assess patients, although sometimes wheelchairs are used by other patients. (S2–10, OTL, F, 10/10/17)
	They (the church) refer their patients to ward-based outreach teams. In turn, we assist or refer to the clinic. (S1–9, OTL, F, 03/10/2017)
	Due to lack of office space all support groups are conducted there. (S1–9, OTL, F, 03/10/2017)

CHW, community health worker; OTL, outreach team leader; FM, facility manager; WBOT, ward-based outreach teams; NGK, Dutch Reformed Church; DOT, directly observed therapy.

#### Individuals, families and communities

Participants described their relationships with the individuals, families and communities as generally positive. These relationships, however, are not automatic or easy to achieve. When CHWs go to people’s homes, they often have to manage negative attitudes and behaviour as well as general service expectations. Teams said that they tried to reduce resistance by working with local authorities and ward committees as well as the clinic committees, where they are represented usually by the WBOT coordinator or facility managers. They also said that they overcame barriers through perseverance and persuasion as well as by delivering services that people need.

#### Health and other service providers

In terms of WBOT functioning in the public healthcare service in Gauteng, WBOT coordinators provide direct active support to teams and team leaders. Teams reported that coordinators routinely assisted with service and training, as well as when they had challenges in their communities or in the workplace. As WBOTs are clinic based, their relationships with facility managers and staff are particularly important. Teams said that they were given equipment and participated in monthly facility meetings and training sessions.

However, they also faced several critical organisational and relationship challenges. Participants reported organisational tensions between clinic and community service priorities that influence their work. Multiple reporting lines meant that facility managers struggled with their dual responsibilities and some acknowledged that they were often not available. At the same time, team leaders and CHWs felt under-supported or, as one CHW put it, supported ‘only for clinic responsibilities’.

Outreach team leaders and CHWs felt that facility management and staff failed to take account of their work. Their poor understanding of and disrespect for what WBOTs do were evident in practical interactions. Team leaders are expected to work in the clinic and with WBOTs in the community. Outreach team leaders and CHWs are often substituted willy-nilly for absent staff. At facilities, WBOT work is not fast-tracked, although OTLs said that they are helped with equipment and patient treatment and do not have to queue to collect medications. They also reported insufficient back referral and a lack of clarity on the referral flow. A facility manager (WR5, facility manager, female, 04 October 2017), for example, said she did not know ‘which flow to use’ to back refer to specific CHWs.

Organisational privileging of facilities over WBOTs is also expressed in OTL and CHW terms of employment. As part of clinic staff, OTLs said they interacted routinely with nurses and sporadically with family physicians at clinics or during training. By contrast, CHWs, who are not part of the clinic staff, said that they are treated differently and made to feel marginal even though many had good relationships with individual clinic personnel.

Within facility services, WBOTs have a strong, but fairly one-sided relationship with the human immunodeficiency virus (HIV) and acquired immunodeficiency syndrome (AIDS), sexually transmitted infection (STI) and tuberculosis (TB) (HAST) vertical program.

Outside of primary healthcare clinics, the WBOTs’ strongest working relationships are with social development and related ‘third sector’ non-governmental organisation (NGO) and faith-based organisation (FBO) welfare partners. Social workers were described in the PLRs as key integrators. Through NGO and FBO partnerships, CHWs and OTLs said they help families and individuals get assistance with grants, blankets and food parcels. They also make and receive referrals and ensure clients get home-based and rehabilitative care. Partner organisations in some places also allow them to use their facilities to run support groups and training.

The study participants said that the relationship of WBOT with local businesses was limited. In Site 1, examples were given about local tuckshops that offered bread and other drinks during events, and an undertaker who allowed CHWs to use his or her venue to give health talks. In Site 2, WBOTs involved local tuckshops in condom distribution.

### Infrastructure and functional equipment

All teams faced basic infrastructural and functional equipment constraints. Space was a major constraint everywhere. The lack of workspace affected the ability of teams to meet, supervise and support workplace learning. There were limited or no resources for photocopying and often they did not have filing and storage space.

The then Gauteng Department of Health requirement that CHWs clock in and out at facilities was a challenge. Participants said that daily they lost time walking to and from the facility. Their work was further disrupted when there were electricity outages because they had to wait to clock in, in order to be paid for the day’s work.

In terms of equipment, all teams were equipped with tablets to support electronic data collection and service delivery. In field, however, it was observed that a number of CHWs did not have paper-based referral forms and had to verbally refer clients without supporting documentation (Peer Exchange Workshop, 12/10/2017). They lacked essential equipment needed to provide community-based healthcare, including blood pressure machines, masks, urine dipsticks, glucometers, gloves and information leaflets. They were also not provided by the system with uniforms, kitbags and name tags. At Site 2, these were eventually obtained by the WBOT coordinator through once-off assistance from a non-governmental partner working in the area.

### Learning in the workplace

Continuous learning to support capacity development using a capability approach is structured into the routine of COPC. Learning sessions were routinised for all teams. They were held either weekly or fortnightly during their Friday management meetings and additionally when there was wet weather. They describe having covered a range of topics (Table 4).

**TABLE 4:** Learning in the workplace.

**Learning practice**	Every morning we come together for a briefing. Then we go out for household registration. On Friday, it’s a teaching and learning day. There is no going out. (S1–9, CHW, F, 03/10/2017)	
	On Friday and in rainy weather we do in servicing. (S2–9, OTL,F, 11/11/2017)	
	Every Friday (we) meet for in-service training, give updates and report on progress and challenges experienced. (S2–2, CHW,F, 10/11/2017)	
	We revise the manual for Phase 1 and we remind each other. We share of our experiences in the households and discuss. Guidelines are read and explained. Lessons are prepared at home. Each CHW gives in-service to others. (S1–9 OTL, F, 03/10/2017)	
**Attitudes and experiences**	I am confident and am able to stand in front of people. I can confidently screen malnutrition and diabetes. (S1–3, CHW, F, 03/10/2017)	
	I have been empowered. I am able to practice what I have learned personally, (with) my family and I am living a positive lifestyle. (S2-3, CHW, F, 11/10/2017)	
	It has boosted my ego. Learning has improved my work. (S1–11, OTL, F, 05/10/2017)	
	CHWs have an idea of how to approach health which is the skills that I have taught them. (S1–4, OTL, F, 04/10/2017)	
	It changed my behaviour and my attitude. (S1–9, CHW, F, 03/10/2017)	
	I am confident. There were things I did not know. I can educate the community. I am able to capacitate others. (S2–2, CHW, F, 10/10/17)	
	Learning makes my work easier. Being able to assist our households in health-related issues and social problems. (S1–9, OTL, F, 03/10/2017)	
	Training on diabetes increased my knowledge and therefore I am able to educate the community, my family and able to give talks on diets and treatment. (S2–3, CHW, F, 11/10/17)	
	(It) empowers the team to be able to tackle all health issues. They are able to teach the community. (S1–4, OTL, F, 04/10/2017)	
	Peer education has improved. Everyone has gained more knowledge. (S2–7, CHW, F, 10/10/2017	
	(I am) learning how to work with people of different cultures, norms and values. (S2–5, CHW, F, 11/10/17)	
**Challenges**	We have been given skills; however, we do not have equipment to do practicals. (S2–3, CHW, F, 11/10/17)	
	In-service training is done repeatedly on the same conditions. (S2–5, CHW, F, 11/10/17)	
	Information has to be given on time. Our team leaders need to be more learned. (S1–9, OTL, F, 03/10/2017)	
	The challenge is the language barrier for some of them during training. We constantly have to have an interpreter. Sometimes time is not on our side due to competing priorities. (S1–5, FM, F, 04/10/2017)	
	Some CHWs want to learn, some are just here for money. (S2–8, OTL,F, 10/10/2017).	
**Participants’ recollection of learning topics covered**		
**Site 1**		**Site 2**
AitaHealth™ (ICT), Referrals, Follow-Ups TB, HIV, PMTCT, Ante- and Post-Natal Care, IMCI Immunisation, Deworming, STIs, Hand Washing, Diabetes, Hypertension, Healthy Lifestyle, Epilepsy, Domestic Violence, Malnutrition, Cancer, Asthma and Malaria, Code of Conduct.		AitaHealth™(ICT), Ante-Natal Care, Post-Natal and Infant Care, Road to Health, Immunisation, Deworming, Vitamin A, Teen Pregnancy, Mental Health, Hypertension, Diabetes, Stroke, Speech Therapy, Hygiene, Healthy Lifestyle, First Aid, Nutrition, Medical Male Circumcision, Integrated Treatment Adherence.

CHW, community health worker; OTL, outreach team leader; STI, sexually transmitted infection; IMCI, integrated management of childhood illness; PMTCT, prevention of mother to child transmission; ICT, information and communication technology; TB, tuberculosis.

There was a generally positive attitude to learning. Participants found learning personally rewarding and a source of pride. They said that the knowledge and skills they had gained made it easier for them to support households. They also said peer learning benefitted everyone. It had helped improve communication between team members, assisted them with their work and helped improve their tattitude towards one another. They also felt that it had helped them relate better to people in general.

Participants reported that IT gave them a better understanding of both service and learning needs. Facility managers and team leaders said they used screening data collected on AitaHealth™ to assist them in identifying the focus of training, while CHWs said the app helped them learn what to ask in different conditions. Teams described the technology as ‘empowering’ and that it had helped improve their individual and collective knowledge of diseases. Although the app guided practice, it was also recognised that team members needed to be supported to link new knowledge with the job process. As one facility manager observed (04.10/2017), ‘things constantly change’ and CHWs need to ‘interpret how to intervene as most of it is not only health related’.

The participants pointed out several real learning challenges. In terms of content, it was generally recognised that more training is needed and that there is a need to provide teams with relevant equipment to practise skills that they are trained in. The participants said that they wanted OTLs to be upskilled, more topics to be covered and that topics should be covered in greater depth. In terms of communication, there was a language challenge that affected learning.

In the field, it was observed that CHWs’ understanding of doing COPC through WBOTs needed to be deepened. Amongst other things, some found it difficult to explain why they register households and the informed consent process. They found it hard to ask some of the questions, for example, around HIV and AIDS and income, as well as to deal with refusals. They also were inclined to provide vertical rather than generalist services. And they needed more training on handling medications in the field to maintain the cold chain. Team leaders also needed timely preparation with new information in order for them to effectively support learning in their teams (Peer Exchange Workshops).

Other challenges in learning that participants identified relate to the absence of certification, the low ‘levels of education’ of some CHWs, unfamiliarity with technology and individual variation in motivation to learn.

### Activities, service organisation and supervision

#### Activities

In a COPC approach, the purpose of the WBOTs is to provide comprehensive care appropriate to the level of service. Amongst the tasks required from CHWs is the registration of households and household triage, a screening process to engage with and respond to healthcare needs from the initiation of service contact.

Table 5 shows the extent of the community-based work collected on AitaHealth in the month preceding the peer review.

**TABLE 5:** AitaHealth™ ward-based outreach team service performance in the 4 weeks (03–28 September 2017).

	Site 1	Site 2
Number of teams	8	8
Number of CHWs	63	58
Households registered	911	1168
Household assessments completed	829	1099
Household triages completed	826	1096
Persons registered	2632	3813
**Follow-ups**		
Follow-ups scheduled	468	1311
Follow-ups completed	103	637
**Triage**		
1. Emergency	1	0
2. TB: On treatment	4	8
3. TB: Completed treatment in past 12 months	20	12
4. TB: Diagnosed and not on treatment	1	1
5. TB: Defaulted on treatment	1	0
6. TB: Showing symptoms	49	14
7. Requires home-based care	3	1
8. Pregnant	22	21
9. May be pregnant	6	4
10. Post-natal care	6	8
11. Chronic conditions	323	417
12. HIV – Requested test	24	6
13. HIV under 18m PCR	21	5
14. U5/Not immunised	107	31
15 Harmful substance use: Health and social problems	20	4
16 Harmful substance use: Inject drug use	5	1
17 Harmful substance use: Household support needed	7	2

CHW, community health worker; HIV, human immunodeficiency virus; TB, tuberculosis; PCR, polymerase chain reaction test.

The participants said that in their community work they predominantly focus on health promotion and disease prevention, giving advice and making referrals to the clinics as needed. They also do treatment support and, to a lesser extent, palliative care. Team leaders and CHWs also worked at clinics. They said that they assist with triage, take vital signs and fetch files there. Teams also do clinic-related work in the community, particularly following up on patients who do not come for TB treatment.

#### Service organisation and supervision

Community health workers work in pairs for safety reasons. They are supervised and supported by OTLs. In turn, they and their teams are managed and supported by WBOT coordinators. Team leaders were described as being ‘hands on’. They hold routine morning meetings. They accompany CHWs on supervised visits. They routinely run team management meetings, usually on Fridays. And they attend monthly team leader meetings with the WBOT coordinator (Table 6).

**TABLE 6:** Services – Data use, activities, organisation and management.

Data to support planning, service delivery and monitoring	
**AitaHealth™**	It’s easy, for example, if children are not immunized, it’s easy to see. (S1–3, CHW, F, 03/10/2017)
	(It) assists by reminding of me of follow-up visits, and therefore I have never missed any dates. (S2–3, CHW, F, 11/10/2017)
	The CHW is able to schedule visits and (be) reminded by the gadget. The OTL is able to make the necessary follow-up with nurses. (S1–5, FM, F, 04/10/2017)
**Organisation and supervision**	(I) meet with them every day, in the morning before they go out. Also I use WhatsApp group to communicate. (S1–2, OTL, F, 03/10/2017)
	Every morning we debrief. (S2–10, OTL, F, 10/10/2017)
	In the morning they share their findings and challenges. (S2–4, OTL, F, 10/11/2017)
	Usually, a supervisor accompanies us to monitor and help address challenges. (S1–3, CHW, F, 03/10/2017)
	Anytime the TL wants to go out they do so. There is no specific day. (S2–8, OTL, F, 10/10/2017)
	Every Friday (we) have weS2-ly meetings to address challenges and progress. Feedback assists (us) to improve in areas of weakness. (S2–2, CHW, F, 10/10/17)
	If they omit to handle screening for TB they can be infected. If they forget to wash their hands they can be infected. (S1–5, FM, F, 04/10/2017)
	They inform the team leader immediately when mistakes happen. They also use a WhatsApp group to address urgent matters. The team leader has a one-on-one meeting or a group meeting with the team. (S2–3, CHW, F, 11/10/2017)
	When there are delays, the team leader addresses them with facility manager and the CHW talks with clients. (S1–3, CHW, F, 03/10/2017)
**Community – Facility**	Most of the time we refer cases to the clinic and different stakeholders. (S1–10, OTL, F, 04/10/2017)
	(I) Write out referral notes almost daily. Household members respond better to referral notes and visit the clinic. (S1–6, CHW, M, 04/10/2017)
	(I) assess the situation and take history, then fill in referral forms to the clinic for further management. (S1–9, CHW, F, 03/10/2017)
	We link the community with the clinic, tracing patients for the clinic, delivering medication, running wellness campaigns. (S1–6, CHW, M, 04/10/2017)
	(I) assist at the clinic in retrieving files for the patients who come for follow-up. (I) assist in distributing medication outside the facility. (S2–1, CHW, F, 10/10/2017)
	Other days we work in family planning. We relieve where there is shortage. (S2–5, CHW, F, 11/10/17)
	After two (o-clock) team leaders work in the clinic. They cannot do their administration work if they are sitting in front of a gadget. (S1–9, OTL, F, 03/10/2017)
	WBOT staff work most of the time in a clinic. (S2–7, CHW, F, 10/10/2017)

CHW, community health worker; OTL, outreach team leader; FM, facility manager; WBOTs, ward-based outreach teams; TL, team leader; TB, tuberculosis.

The participants reported that team leaders encouraged CHWs to have good work ethics and to work as a team, share responsibilities and build each other. Amongst other initiatives, OTLs rotate partners through teams to enable CHWs to share information, skills and experience. They also encourage CHWs to apply what they have learnt to themselves as well as to the people in the community.

Team members were actively made aware of possible future consequences of their practices, both through education and advice as well as through peer group pressure and formal disciplinary processes. When there are mistakes, CHWs said that they reported these to the OTLs. Team leaders either dealt with them directly or sought guidance from facility managers or WBOT coordinators.

Community health workers and OTLs are exposed routinely to distressing situations. In the field at both sites, it was observed that there was insufficient debriefing.

### Recommendations and forward planning

In the peer review process, the purpose of the peer-learning exchange workshop is to generate a shared understanding of issues and to make recommendations and plans to improve practice. At both sites the teams generated practical recommendations that related to operational issues, work integrated learning and support (Table 7).

**TABLE 7:** Peer exchange workshops – Recommendations and forward planning.

Site 1	Site 2
Operational	Operational
Shortage of meeting and learning space – additional structures (health posts) needed.	Raise shortage of meeting space at district level with operational and facility managers.
AitaHealth™ should be used to clock in and out.	Address referral issues – processes, forms, capture of activities.
Team leaders to concentrate on WBOT only.	AitaHealth™ should be used to clock in and out.
WBOTs need the support of professional nurses.	CHWs should refer emergency cases to the emergency department (ambulances).
	Gadget problems should first be addressed to district IT, then upwards.
	The gadget must only be used for work purposes to extend battery life.
	In case of emergency CHWs should send a call back to the team leader.
**Work integrated learning**	**Work integrated learning**
10 day training for CHWs and OTLs to be reviewed.	To focus on health promotion on and disease prevention.
To focus on health promotion on and disease prevention.	Learning to be led by team leaders, but CHWs can be also be allocated topics to prepare and lead.
The peer-learning review to be repeated in 4 months. All clinics have two teams – they can review one another.	The peer-learning review to be repeated in 4 months. All clinics have two teams – they can review one another.
**Tasking for teams over next 4 months**	**Tasking for teams over next 6 months**
Community-oriented primary care should be highly emphasised.	Strengthen relationships with all relevant local institutions.
As first-line contact CHWs need to be empowered because they liaise between the health services and the community.	Link to community policing forum – to support CHWs working in dangerous areas, like hostels.
Focus on health promotion during service delivery.	
Focus on LISA documentation and building partnerships.	

CHW, community health worker; OTL, outreach team leader; IT, information technology; WBOTs, ward-based outreach teams; LISA, local institutional support assessment.

As part of forward planning, both sites then agreed on common tasks that all teams could action over the next 6 months. Site 1 WBOTs prioritised health promotion during service delivery and the improvement of LISA documentation, including the inclusion of LISA forms on AitaHealth™. Site 2 WBOTs prioritised strengthening relationships with all relevant local institutions, especially with the community policing forum in order to support CHWs working in dangerous areas, like hostels.

## Discussion

As a methodology, PLRs apply participatory action research to understand and support implementation fidelity and encourage quality improvement processes. The results show that PLRs are able to generate a rich and multi-layered understanding of what it means to deliver healthcare services to people in their homes in the existing public primary health system. They are practically helpful for participants because they are structured to support inclusive, context-specific learning for everyone involved in the delivering of services, wherever they are located along the care pathway.

Peer-learning reviews are also of value to others. The benchmarks that guide the process are derived from assumptions about how WBOTs are expected to work and what they are supposed to do. The results show both the achievements and the challenges of making these assumptions reality. Particularly, they show how important context is to understanding why plans do not translate in linear and literal ways into practices. Thus, while mapping to define CHW and WBOT areas of practices may appear from above to be ‘straightforward and once off’, the view from below shows place and space as fluid, something to be navigated, something to be traversed many times. Similarly, the structural privileging of facility-based services in public primary healthcare organisationally marginalises CHWs and the work that WBOTs do. This is why it is possible for them to be taken out of the community to work in facilities and simultaneously not to be considered as facility staff, or to be expected to meet facility and patient needs without being properly equipped. The achievements of these teams show that the essential cooperation needed between levels of workers to ensure basic patient healthcare is hard won in a bureaucratised institutional hierarchy.

The PLR process and the implementation results demonstrate the importance of structuring learning into service and research. Both helped develop healthcare professionals’ and workers’ abilities to better understand what they do, do their work, grow their sense of self-worth and improve their relationship with others.

The study shows that PLRs make the practical issues and experiences of service delivery visible to all team members. And as elsewhere,^10^ the PLR processes of partnering and collectively reflecting on issues were found to be non-threatening, supportive of cooperation and encouraging of learning as an intrinsic part of doing. Individually and collectively they created an opportunity for participants to improve their own and others’ healthcare abilities.

## Limitations

The envisaged PLR process had to be modified because of meeting space and service demand constraints. These impacted inclusivity as not all facility managers and WBOT members were able to attend the peer exchange workshops. This may have led to the omission of some important issues or perspectives that would need to be picked up in future reviews. Also, although every effort was made to ensure that PLR teams included members with relevant experience and skills sets, practically variations in both these dimensions may have influenced the reporting and feedback received from each team. It is expected that individual and collective capacity to do PLR will improve with practice. The data presented reflect the outcome of the PLR process itself. Further research is needed to determine whether PLRs translate into improved practices. Lastly, doing ICT-enabled COPC is one of the many approaches to WBOT implementation in South Africa. This said, the principles, framework and processes of PLR can be applied to other applications and contexts in Africa and elsewhere.

## Conclusion

The PLR methodology produces an intense experiential learning and self-monitoring process in which peers and colleagues engage with their work. As a facilitated process that is scaffolded by a benchmark guide that covers key deliverables as well as purposive interactions, it gives participants an overview of the work they do. They gain insight into their achievements and challenges and find possible solutions to improve their own well-being as well as their contribution to service delivery and individual, family and community health. As an ‘own account’, the PLR reveals the imperfect but substantial practice of community-based healthcare that can be achieved by ICT-enabled COPC delivered by WBOTS.

Amongst the lessons learnt from implementing the PLRs are that they need to accommodate local contexts, they should be scheduled into the annual district calendar at intervals of about 6 months and they need to be budgeted for financially and organisationally to ensure that venues, transport and refreshments enable inclusivity. In this way it is possible to maximise the benefits of PLRs without creating excessive demands on time and other resources.
